# Precision Nutrition and Cancer Relapse Prevention: A Systematic Literature Review

**DOI:** 10.3390/nu11112799

**Published:** 2019-11-16

**Authors:** Clara Reglero, Guillermo Reglero

**Affiliations:** 1 Institute for Cancer Genetics, Columbia University Medical Center, New York, NY 10032, USA; 2 IMDEA Food Institute, 28049 Madrid, Spain; guillermo.reglero@imdea.org; 3 Institute of Food Science Research (CIAL), Autónoma de Madrid University, 28049 Madrid, Spain

**Keywords:** precision nutrition, cancer relapse, bioactive phytochemical foodstuffs, lung, breast, prostate, colon, leukemia

## Abstract

Cancer mortality rates are undergoing a global downward trend; however, metastasis and relapse after surgery and adjuvant treatments still correlate with poor prognosis and represent the most significant challenges in the treatment of this disease. Advances in genomics, metabolomics, and proteomics are improving our understanding regarding cancer metabolic diversity, resulting in detailed classifications of tumors and raising the effectiveness of precision medicine. Likewise, the growing knowledge of interactions between nutrients and the expression of certain genes could lead to cancer therapies based on precision nutrition strategies. This review aims to identify the recent advances in the knowledge of the mechanistic role of bioactive phytochemicals in foodstuffs in tumor progression, metastasis, and chemo-resistance in order to assess their potential use in precision nutrition therapies targeting relapse in lung, breast, colon, and prostate cancer, and leukemia. A considerable number of bioactive phytochemicals in foodstuffs were identified in the literature with proven effects modulating tumor growth, progression, and metastasis. In addition, the use of foodstuffs in cancer, and specifically in relapse therapies, is being reinforced by the development of different formulations that significantly increase the therapeutic efficiency of these products. This can open the possibility for testing combinations of bioactive phytochemicals with cancer relapse treatments as a potential prevention strategy.

## 1. Introduction

### 1.1. Breast, Colon, Lung, Prostate and Leukemia: The Deadliest Cancers

Lung, breast, prostate, and colon cancers have the highest overall incidence and represent 36.4% of the total diagnosis according to GLOBOCAN 2018, a report that gathers data from the International Agency for Research on Cancer of the World Health Organization [1]. Besides their high occurrence, these types of cancer add up to a mortality rate that reaches 49.2% of the diagnosed cases. In addition, blood malignancies count with a rate of 6.5%, an incidence particularly worrying as it is the highest in children and young adults.

In the last five years international scientific production related to new basic and translational discoveries on cancer has grown by 5% annually. Lung, colon, breast, prostate, and leukemia cancers account for 51% of scientific publications on cancer in this period (Web of Science, Clarivate Analytics, Philadelphia, PA, USA). Many papers address the association between gene regulation and cancer progression or metastasis, still representing the main approach in cancer research. More recently, an increasing number of investigations based on genomics and epigenomics are focusing on the improvement of tumor classification, the relationship between cancer and microbiota, or the discovery of new therapeutic targets in metabolism, in order to develop specific therapies in the context of precision medicine.

#### 1.1.1. Lung Cancer

Survival is the foremost concern in lung cancer due to its progressive metastasis and resistance to therapy and, furthermore, the molecular hallmarks of its malignant properties are still insufficiently known. Recently, a 17-gene panel involving critical cellular processes such as hypoxia-mediated epithelial-mesenchymal transition (EMT) or epigenetic modifications was identified as a potential biomarker tool for prediction of metastasis and prognosis in patients affected by non-small-cell lung cancer (NSCLC) [2]. Other example of the last advances in this field is the detection of the scaffold WT1-interacting protein (WTIP) as a novel tumor suppressor down-modulated in NSCLC. Lower levels of these proteins significantly correlate with poor prognosis as a result of higher rates of cell proliferation and tumorigenesis [3]. Additionally, increasing findings in immunotherapy open expectations for the improvement of the prognosis of lung cancer patients [4].

#### 1.1.2. Breast Cancer

Regarding breast cancer, tumor heterogeneity makes patient classification according to their risk of metastatic relapse essential in order to guide decisions in adjuvant chemotherapy. However, in the era of personalized medicine, genomics alone does not provide enough knowledge to apply precision treatments to specific tumors. Therefore, complementary multi-omic studies are emerging as the most promising approaches addressing breast cancer complexity [5]. For instance, a quantitative proteotyping approach based on sequential windowed acquisition of all theoretical fragment ion spectra mass spectrometry (SWATH-MS) has been proposed to establish key proteins for breast tumor classification [6].

#### 1.1.3. Prostate Cancer

The application of new technologies now allows progressing in the understanding of the molecular basis of prostate cancer pathogenesis. Microarray-based transcriptomic analyses and next generation sequencing technology are examples of powerful tools for studying gene expression and changes in gene structure at the transcript level. Recent publications described new mechanisms regarding tumor suppressors in this type of tumors [7] and new potential therapeutic targets [8]. Also, these technologies played a critical role in recent studies indicating specific gene fusions, present in at least 50% of prostate tumors, as key modulators of gene expression promoting tumor growth and progression in prostate cancer [9].

#### 1.1.4. Colorectal Cancer

Deregulated cellular energetics is another of the hallmarks of cancer. In this sense, cooperative lipid metabolism-related genes involved in colorectal cancer progression have been recently identified. The acyl-CoA synthetase/stearoyl-CoA desaturase (ACSL/SCD) lipid network fuels migratory and invasive properties through EMT induction and is associated with an increased risk of relapse in colorectal cancer patients [10]. Other molecular mechanisms associated with the refractory nature of cancer point to cancer stem cells (CSCs). In this regard, it has been recently published that proliferation of CSC-enriched colon spheroids is dependent on mTORC1 kinase, which is activated by reactive oxygen species (ROS) produced by an NADPH oxidase [11]. Furthermore, the potential correlation between gene polymorphisms and colon cancer progression continues being studied and validated in order to identify prognostic indicators supported by mechanisms such as the increased invasiveness of tumor cells through the expression of genes involved in the activation of fatty acids through conversion to acyl-CoA [12], or the regulation of COX2 expression and cell apoptosis [13]. On the other hand, it has been recently discovered that microbiota plays an important role in colorectal carcinogenesis. This opens up new opportunities for using microbiota profiling information in colorectal cancer prevention, diagnosis, and therapy [14].

#### 1.1.5. Blood Malignancies

Recent advances in leukemia genomics and epigenomics have facilitated the study of clonal populations and their genetic-epigenetic evolution, changing the classic view of leukemia into a complex heterogeneous disease aggravated by clonal evolution [15,16]. These findings allow a better understanding of the mechanisms involved in leukemia transformation and therapy resistance and reinforce precision medicine as the most promising approach to leukemia treatment. The complexity of this disease, together with the high number of patients showing chemotherapy resistance, evidences the urgent need of more efficient therapies. In the last years, new therapeutic approaches are being investigated, as for instance monoclonal antibodies-based treatments [17] or therapies based in macrophage targeting [18].

### 1.2. Cancer Relapse

Cancer relapse worsens the prognosis of patients and is a factor that contributes significantly to mortality. However, only 2% of cancer publications in the last five years deal specifically with relapse (Web of Science, using databases including MEDLINE). Cancer recurrence involves many biological interactions, such as genetic, transcription, environmental, endocrine signaling, and metabolism. These interactions add another layer of complexity in the understanding of cancer recurrence and metastasis, delaying progress in therapeutic opportunities [19]. Lung cancer and leukemia mortality rates are mainly due to their higher recurrence rates, compared to breast, colon, and prostate, for which surgical resection of the tumor combined with adjuvant treatments accomplish higher survival rates [20].

Lung cancer has a recurrence rate that reaches 50% of patients. Despite having noteworthy advances in recent years on the knowledge of the disease biology and mechanisms of tumor progression, the survival rates remain low and more research is necessary on molecular aspects that give rise to more effective therapies [21]. Discovering the association of gene expression with recurrence is of utmost interest in order to develop therapies to prevent and treat relapse [22]. For example, it has recently been identified that the long non-coding RNA AWPPH over-expression is involved in NSCLC recurrence by the upregulation of TGF-b1, finally increasing cancer cell migration and invasion [23].

In respect of leukemia, the high number of relapse-specific mutations acquired by patients affecting genes involved in different functions, leading to clonal evolution and resulting in chemotherapy resistance, points to the high molecular complexity of this process [24,25]. Understanding the role of relapse-specific mutations will foster the development of new therapies for the treatment of high-risk patients. In recent years, several groups have dealt important advances in the knowledge of these mutations using whole-genome sequencing technology in leukemia patient samples. For instance, mutations in the phosphoribosyl pyrophosphate synthetase 1 gene (*PRPS1*), encoding a purine biosynthesis enzyme, were associated a few years ago with chemotherapy resistance and relapse [26]. In addition, the role of the cytosolic 5’-nucleotidase cytosolic II gene (*NT5C2*) has recently been described regarding clonal evolution and chemotherapy resistance, with important implications in the development of new targeted inhibition therapies [27,28].

In essence, the recent discoveries in relation to drug resistance and previously unknown molecular mechanisms associated with metastasis and recurrence demand alternative therapeutic strategies, among which metabolism could emerge as a new cancer therapy support. Although cancer relapse is due to a multitude of genetic mutations and biochemical processes, advances in genomics, metabolomics, and proteomics allow better understanding of the metabolic diversity due to genetics and microbiome variation, as well as a detailed classification of tumors, which provide precision medicine with individual treatments. Likewise, the best understanding of the metabolic variation allows us to know the interactions between nutrients, metabolism, microbiota, and related genes, facilitating the development of adjuvant cancer therapies based on precision nutrition strategies [29].

### 1.3. Precision Nutrition and Cancer Therapy

Over the years, numerous epidemiological studies have been carried out to link the diet with cancer, either from a preventive approach or by associating the consumption of certain food products with tumor generation and growth. However, in parallel to the development of precision therapies in medicine, precision nutrition is an emerging science that relies on well-established factors such as genetic and epigenetic variation [30] and the microbiome [31]. It has recently been shown that the treatment of human cell lines with different bioactive foodstuffs influences their physiological attributes depending on their ability to influence the expression of different genes [32]. The possibility of using nutritional therapies against cancer, as a complementary medicine, is internationally accepted due to its advantages of less toxicity and better acceptance by patients [33]. In the case of breast cancer, complementary phytochemical therapy in adjuvant treatments has been proposed both with preventive effects and during conventional treatments after diagnosis [34], concluding that nutritional strategies can be effective for prevention of relapse [35].

Epidemiological studies triggered further research in terms of molecular mechanisms, which have significantly improved the effectiveness of phytotherapy, entering the context of precision nutrition [36]. Recent studies on the association between prevention, treatment, and recurrence of cancer suggest the benefit of investigating the link between specific food components and certain health outcomes [37]. Since very specific therapeutic targets must be reached, precision nutrition must be based on individual foodstuffs with well-established mechanisms of action at the molecular level in terms of gene expression modulation and signaling pathways involved in proliferation, invasion, angiogenesis, and metastasis or apoptosis [38]. For example, it has been shown that it is possible to attack genetic instability associated with cancer through nutritional strategies that inhibit proliferative signaling, attenuate oncogenic metabolism, and block inflammation [39].

The biological activity of food polyphenols, a broad family of compounds with representatives in virtually all foods, has been specially studied for decades since they have in common an intense antioxidant activity that suggests other potential health outcomes, for example in breast cancer [40]. Recently, intensive research has been carried out to determine the preventive or therapeutic activity of different natural phenolic compounds [41], opening ways for its application in new treatments of various types of cancers such as breast [42,43,44,45], colon [46], or prostate [47,48]. Some synthetic phenolic compounds have also been successfully studied for the treatment of some cancers [49]. In addition to polyphenols, curcumin (diferuloylmethane) is one of the most studied foodstuffs in recent years as a potential therapeutic product for cancer and more specifically for leukemia [50]. Traditional food products, such as rosemary extract, have been proposed as potential ingredients of precision nutritional supplements in cancer therapy, identifying molecular mechanisms related to the effects and the interactions with currently-used anticancer agents [51]. In the case of colorectal cancer, lipid-metabolism-related genes have acquired relevant interest for precision nutrition therapies, since a wide range of tumorigenic steps can be influenced by lipid metabolism, both in primary tumors and distal metastasis [52]. Certain therapeutic strategies based in diet patterns during adjuvant treatments are also sometimes considered as precision therapies [53]. Finally, multi-targeting profiles of food ingredients are being investigated regarding their potential roles triggering anti-cancer molecular mechanisms through the modulation of certain gene expressions or signaling pathways [54].

The objective of this review is to identify the recent advances in the knowledge of mechanisms that support the effects of bioactive phytochemicals in foodstuffs associated with molecular targets of tumor progression, metastasis, or chemo-resistance, in order to assess the potential use of these products in precision nutrition therapies addressing the prevention of relapse in lung, breast, colon, and prostate cancer, and leukemia.

## 2. Material and Methods

### 2.1. Systematic Search

A literature search was carried out on September 30th 2019 in Web of Science using the following databases: Web of Science Core Collection (WOS), Current Contents Connect (CCC), Derwent Innovations Index (DIIDW), Korean Journal Database (KJD), MEDLINE, Russian Science Citation Index (RSCI) and Scientific Electronic Library Online (SCIELO). In order to capture only the most recent advances, articles published between 2017 and 2019 were considered. The language filter was English. The same search strategy was carried out in SCOPUS. PUBMED was not used since it is a search engine in MEDLINE, already included in WOS.

Search a strategy included three layers of keywords: (1) types of cancer targeted; (2) the most studied bioactive foodstuffs in the literature cited in the introduction, together with keywords indicating the characteristic factors of precision nutrition; (3) keywords identifying cancer relapse trying to cover all synonyms. In order to open the search, asterisk-terminated keywords were introduced. The search for the first indicator (the five target types of cancer) was performed on the “title” field of the search engine. However, the search for the second indicator, the one related to bioactive foodstuffs and the third one related to cancer relapse and its synonyms, were searched in the “topic” field, which includes title, keywords, and abstract; in order to include all types of research approaches. The keywords were combined using boolean operators “AND”, “OR”. The detailed search strategy is presented in Table 1.

The first field included general descriptors of cancer and its scope before “AND”. The second part identified the five types of cancer that are the subjects of this review. The first part of the second field included keywords on bioactive foodstuffs associated in the literature to positive effects on tumor progression or metastasis: Phytochemicals and plant extracts, phenolic compounds of various chemical structures, omega-3 polyunsaturated fatty acids (mainly docosahexaenoic acid (DHA)), and curcuminoids that are being studied in the field of cancer therapies in recent years [55,56]. The second part includes prefixes of concepts that cover the main mechanistic characteristics of precision nutrition studies such as genes, genetics and epigenetics, genomics, microbiota, and microbiome. The third field comprises different denominations of cancer relapse.

### 2.2. Inclusion Criteria

Publications that met the criteria of the search strategy were downloaded in full text and reviewed by the authors. Articles referring to dietary indices for prognosis, epidemiological studies, articles without identification of the specific molecules of the products studied, articles related to food groups and all those not containing a mechanistic explanation of the effect of the bioactive studied, were excluded.

## 3. Results

### 3.1. Literature Search

**Figure 1:**
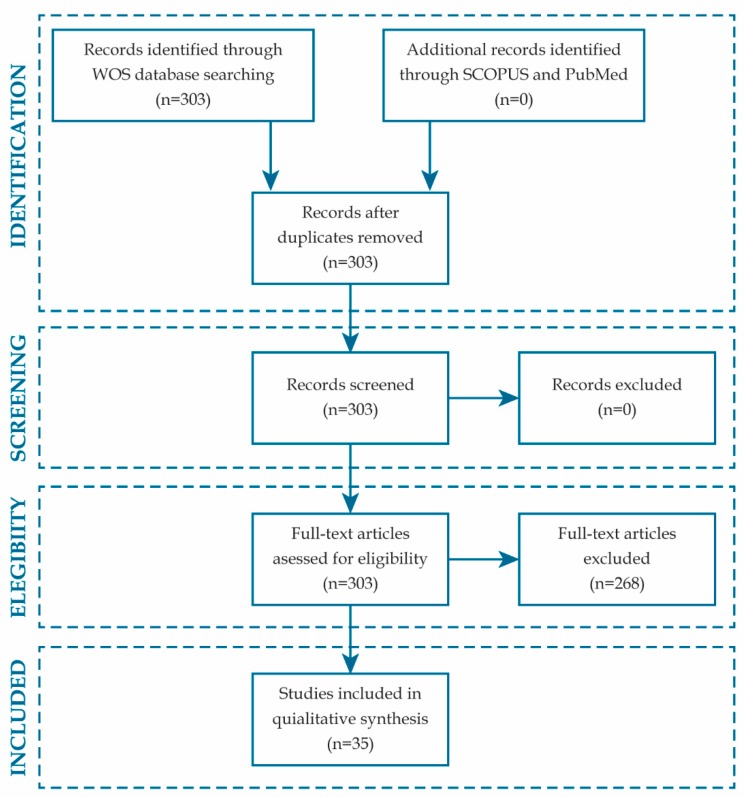
Flow diagram of search and article selection.

Figure 1 shows the flow diagram of the search and article selection process. The search in WOS provided 303 results, while the search in SCOPUS contributed 271. All the articles obtained from SCOPUS were included among the WOS retrieved. In consequence, 303 was the number of records remained after removing the duplicate articles. Full texts of the 303 initially identified papers were revised to assess their eligibility. Literature review articles, epidemiology studies, and papers referring to diets or nutrient groups were excluded. As a result of this revision, 35 articles on phytochemicals or bioactive foodstuffs, containing mechanistic descriptions of their effects on tumor growth, progression, metastasis or, more specifically, relapse, were finally included for qualitative synthesis.

### 3.2. Characteristics of Included Studies

In the initial screening of titles, abstracts, and keywords, no records were excluded since the 303 identified papers met the search criteria. However, the strict evaluation of the full-text articles revealed that most of them did not contain adequate studies for the purpose of this review. Consequently, just over 10% of the papers identified were included for qualitative synthesis. Only research papers including molecular mechanistic knowledge were accepted, according to the objective of this review. Nevertheless, certain research on well-characterized bioactive extracts of plant, fruits, vegetables were also accepted although they did not include new mechanistic contributions.

### 3.3. Outcome

Twenty of the original research studies finally selected for qualitative analysis dealt with polyphenol foodstuffs, and three with lipids, mainly omega-3 and more specifically DHA. In addition, 12 articles on bioactive plant extracts were identified. Table 2 shows, categorized by types of compounds, the main characteristics of the articles that study the molecular mechanisms of individual bioactive foodstuffs with relevance in cancer relapse. 

All compounds in Table 2 are frequently-used food products of which safety and bioactivity have been extensively studied. Overall, these products synergize with chemotherapy treatments by affecting routes associated with cell proliferation, migration or invasion, or by activating apoptosis. Altogether, these effects result in tumor growth repression, finally contributing to cancer remission and relapse prevention.

Regarding research on anticancer effects of food extracts, the knowledge of mechanisms of action is far more limited compared to polyphenol foodstuffs, due to the integration of several compounds in each extract. Despite this, they have been considered in this study since they may tentatively constitute ingredients of nutritional supplements for the treatment of different cancers. Table 3 summarizes the anticancer effects and features of food extracts with known molecular mechanisms included in this review.

### 3.4. Nanotechnology and Precision Nutrition for Cancer

The inclusion of natural bioactive foodstuffs in therapies for different types of cancers is increasingly being accepted in the clinical setting. Potentiating the use of these bioactive compounds in cancer treatment requires improving its bioavailability. Therefore, a significant activity formula development is being carried out in this regard. In this sense, the case of curcumin stands out, since it is one of the most studied bioactive foodstuffs in terms of its potential anticancer effect. Various curcumin nano-preparations have recently been developed. Table 4 shows the latest published works on curcumin nano-formulations that have shown superior antitumor activities than the pure bioactive product.

Formulations aiming to increase the bioavailability of bioactive natural foodstuffs are essential for the application of these products in cancer therapies since, as we have seen, most of the phytochemicals reviewed in this work are generally hardly bioavailable polyphenols. Development of new nano-formulations of bioactive foodstuffs is key to widespread use of these products in cancer therapy.

## 4. Discussion

Since the same factors that are successfully driving precision medicine in cancer serve to design precision nutritional therapies, it is foreseeable that a new era in the treatment of cancer can be opened in coming years. Integration of nutritional strategies may be of special interest for patients treated with adjuvant therapy, in order to enhance therapy effects and to prevent cancer relapse. The knowledge regarding several bioactive foodstuffs mechanisms of action, and the fact that most of them address well established molecular targets, allows the transition into a potential clinical use. In the last decade, a growing number of research works have investigated the anticancer effects of bioactive natural products. However, only very recently have the molecular mechanisms by which nutrients may prevent relapse been explored.

Table 2 includes a list of bioactive food compounds identified in the literature review, and the molecular mechanisms of their anticancer effects. Polyphenols have been the most studied family of compounds in decades for their different biological activities. In addition, some flavonoids have recently shown antitumor and antiproliferative activities that can be useful in relapse-preventive treatments. Epigallocatechin-gallate (EGCG), a flavonoid present in green tea, has been shown to inhibit tumor cell growth and increase apoptosis, promoting tumor suppression. This compound sensitizes human colon cancer cells to 5-fluorouracil, increasing the effects of adjuvant treatment and improving prognosis, and finally reducing tumor relapse risk [68]. EGCG can also prevent lung cancer relapse in lung cancer mouse xenografts by blocking the cancer stem-cells-like growth through the modulation of the hsa-mir-485-5p/RXRα axis and downregulating protein acetylation in lung carcinoma cells [65,66]. EGCG has been also been predicted to affect several pathways involved in cell death and survival, potentially leading to a reduced cancer progression [67]. However, further molecular validations are needed in this sense. Another flavonoid with interesting features for relapse prevention is quercetin, which is present in vegetables such as onions. Quercetin promotes apoptosis and inhibits cell proliferation by modulating important signaling targets as PI3K/Akt or NF-κB effectively eliminating prostate CSCs. It also limits cell migratory capacity and progression of prostate cancer cell lines by the downregulation of MK [71]. In breast cancer, quercetin can help preventing relapse by decreasing expression and activation levels of mTOR, PI3K and Akt proteins leading to a significant inhibition of MCF7 cancer cell proliferation [72]. Another flavonoid with potentially interesting effects for the development of precision nutrition products is apigenin, present in fruits, vegetables, and food herbs such as parsley. This compound improves the effectiveness of adjuvant therapy with cisplatin enhancing both cytotoxicity and its anti-migratory effect on prostate cancer stem cells [57]. Apigenin also helps preventing metastasis and relapse in non-small cell lung cancer cell lines and in an in vivo orthotopic bioluminescent xenograft model by inhibiting cell migration and invasion [58]. Other interesting flavonoids are naringenin, obtained from citrus peel, which has been shown to inhibit proliferation and to induce apoptosis in prostate cancer cells [69], and procyanidin-B2-3,3″-di-O-gallate (B2G2) extracted from grape seeds, that targets both differentiated cells and CSCs leading to tumor mass reduction [70].

Curcumin is another bioactive food product that has been the subject of former numerous studies. Its anti-cancer effects, due its ability to modulate critical anti-apoptotic effectors such as Bcl-xl and NF-κB, are of prominent interest in potential relapse prevention treatments. Moreover, curcumin synergizes with vesicular stomatitis virus (VSV)-based oncolytic treatments modulating antiviral responses and components of the intrinsic apoptotic pathway in a prostate cancer cell model [59]. Furthermore, in colorectal cancer, curcumin can modulate gene expression of *HSPA5*, *SEC61B*, *G6PD*, *HMOX1*, and *PDE3B*, affecting essential pathways like DNA replication or the cell cycle. On the other hand, the synergy of curcumin and oligomeric proanthocyanidins emerges as an opportunity to develop effective therapies, since both compounds share similar molecular mechanisms [60]. Recently, relevant effects of curcumin in breast cancer cell models have been discovered, being that this compound is able to increase the expression of E-cadherin and decrease the expression of mesenchymal markers [61]. It has been also shown that curcumin enhances the effect of some targeted drugs used in cancer such as gefitinib, an EGFR inhibitor, inducing autophagy-mediated apoptosis. This observation opens up opportunities for the use of this compound with treatments to prevent cancer relapse [62].

Some lipid character food bioactives also show anti-cancer effects with mechanisms of action interesting for cancer relapse prevention. In this sense, docosahexaenoic acid (DHA) modulates the growth of colorectal cancer cells and induces expression of genes related to apoptosis [63,64]. Also, β-sitosterol-d-glucoside has inhibitory effects on breast cancer cells growth [74].

The studies mentioned above explain many of the effects on cell growth, tumor progression and metastasis of food bioactive products, opening promising opportunities for the application of these phytochemical foodstuffs in therapies for cancer relapse in breast, prostate, lung, and colon cancer. However, no studies of individual food products were found that applied to leukemia relapse. Regarding this type of cancer, only one paper was recovered, suggesting that additional research should be made in this sense. The cited article analyses the effects of a ginger extract rich in gingerols, demonstrating that this extract exerts a synergistic interaction with methotrexate with high antiproliferative impact in the drug-resistant leukemic sub-lines [78]. Nevertheless, the mechanism that supports this effect is not explained. Indeed, the same happens with other food extracts recently studied and would require additional studies.

Table 3 shows the publications on natural extracts identified in the bibliographic search. In general, interesting antitumoral, antiproliferative, or antimetastatic effects are demonstrated, however, the molecular mechanisms concerning these effects remain largely unknown in most cases. An exception may be the grape seed extract, rich in proanthocyanidins associated with antitumor effects in colorectal cancer in combination with curcumin, by regulating cell cycle and migration [80]. Other extracts with identified effects on cell growth, tumor progression and metastasis in colorectal cancer are watercress extract, rich in phenethyl isothiocyanate [86]; ginseng extract, rich in ginsenoside Rg3 [79]; and isodon extract, rich in flexicaulin A [81]. Importantly, orange peel extract, rich in nobiletin, sinensetin, scutellarein tetramethylether, and tangeretin, exerts a synergistic interaction with 5-fluorouracil in colorectal cancer, modulating EMT transition, inhibiting cell proliferation and modulating cancer stemness, demonstrating a significant potential use of this combination in relapse preventive therapies [82]. In any case, further research needs to be done in order to identify molecular mechanisms regarding the effects of these extracts. Although only individual compounds are now useful in precision nutritional strategies against well-identified therapeutic targets, these studies allow considering the extracts as an intermediate step towards the purification of the specific component responsible for the observed effect. In addition, the low toxicity of these extracts makes them suitable for other support therapies as nutritional supplements.

One of the barriers to be overcome in the application of bioactive phytochemical foodstuffs in cancer therapies is the frequent lack of bioavailability of these products. In this sense, new formulation strategies are being developed, such as bioactive carrier lipids in which the carrier not only increases bioavailability, but also provides a synergistic biological activity with the active ingredient. Interestingly, it has been reported that the use of shark liver oil rich in alkylglycerols as a bioactive lipid vehicle for rosemary extract shows synergistic effects in the expression of genes associated with immune modulation, inflammation, oxidative stress, lipid metabolism, and tumorigenesis in colorectal cancer [84]. Besides this, a good example of a non-soluble and instable product is curcumin. This compound is emerging as a potent effector acting on numerous signaling and molecular pathways that regulate tumor growth and cancer relapse. Despite the growing knowledge about its properties, curcumin cannot be approved as a therapeutic compound due to its limitations in terms of bioavailability and stability. Regarding this, several formulation studies are being performed and a significant number of cases have provided better therapeutic results than the individual bioactive [87,88,89,90,91].

## 5. Conclusions

**Figure 2:**
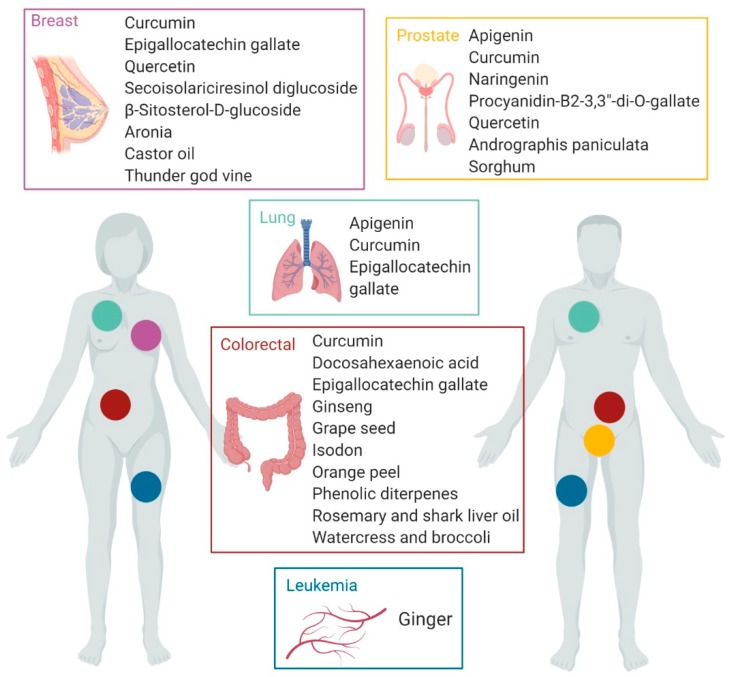
Bioactive foodstuffs and natural extracts with proven effects in cancer treatment. (created with BioRender).

Although, in the context of cancer research, studies that refer to nutritional therapies based on the use of bioactive foodstuffs in adjuvant treatments are still limited, current results are encouraging since there are several phytochemical bioactive foodstuffs with proven modulating effects of tumor growth, progression, and metastasis, and therefore can be tested in humans with a reasonable probability of success if they are applied in cancer relapse treatments (Figure 2). In this sense, the compounds currently identified for this purpose are in general extensively-studied products in the last years such as the polyphenols apigenin, epigallocatechin gallate, procyanidin B2-3,3-di-O-gallate, quercetin, naringenin, secoisolariciresinol diglucoside, and curcumin, as well as docosahexaenoic acid and β-Sitosterol-d-glucoside lipids. Among the targeted cancers included in this review, lung and colon cancer are being widely studied, and prostate and breast cancer have concentrated the largest number of applications. However, there is a great lack of studies related to the use of phytochemical foodstuffs in leukemia therapies and more studies are needed in this disease. The possibilities of using foodstuffs in cancer treatments and more specifically anti-relapse therapies, are being reinforced with the development of formulation technologies that significantly increase the efficiency of these products.

Cancer heterogeneity is one of the features of this disease that makes its treatment particularly challenging. Innovative therapies for patients are continuously being tested and progress has been made in the last years developing early diagnosis protocols and improving patient prognosis. However, despite these efforts, a high percentage of patients relapse after surgery or initial therapy. Cancer relapse involves a great number of different molecular mechanisms that vary form one patient to another. This points to precision medicine as a key element to personalize cancer treatment and prevent relapse, and suggest the value of new effective and safe compounds that potentiate the effects of already-known chemotherapy agents. In this sense, the growing number of studies regarding the mechanisms of several bioactive foodstuffs in the treatment of different types of cancer, open a new layer in precision cancer therapy. Its association with specific genetic targets or different molecular pathways inhibiting tumor growth and metastasis constitutes an important customization component. Moreover, it shows high synergism with several chemotherapy drugs, acting as enhancers of these anti-tumor effects or even sensitizing and reverting chemotherapy resistance. In essence, these products are emerging as novel complementary agents that can be useful in precision nutrition therapies addressing relapse prevention in treatments of cancerous processes.

## Figures and Tables

**Table 1:** Search Strategy.

Field	Keywords	Boolean Operator
**Title**	((cancer* OR carcino* OR tumor* OR tumour* OR onco*) AND (lung* OR breast* OR mammar* OR colon* OR colorect* OR prostat* OR leuk*))	AND
**Topic (title, keywords, abstract)**	((phytochem* OR polyphen* OR flavon* OR gallate* OR catechin* OR omega* OR DHA OR docosahexaenoic* OR terpen* OR curcum* OR extract*) AND (gen* OR genetic* OR genomic* OR microbio*))	AND
**Topic (title, keywords, abstract)**	(relapse* OR recurrence* OR reappearance* OR replication* OR repetition* OR return* OR reemergence*)	

By using prefixes ending in an asterisk, a greater opening of the search is achieved, since keywords are identified more securely even if they end with different plurals or suffixes.

**Table 2:** Characteristics of included studies related to polyphenol foodstuffs. Results are alphabetically sorted by bioactive foodstuff. Molecular mechanisms are summarized indicating inhibition (*↓*) or activation (*↑*) regarding either gene expression, signaling pathways, protein stability, or protein post-translational modifications. Regulated targets are shown in italics.

Bioactive Foodstuff	Source	Cancer Type	Molecular Mechanism	Anticancer Effect	Reference
Apigenin	Fruits Vegetables Food herbs	Prostate	Apoptosis *↓ Bcl-2, sharpin and survivin* *↑ caspase-8, Apaf-1, p21, p53* Signaling pathways inhibition *↓ PI3K/Akt, NF-*κ*B* Cell cycle inhibition *↑ p21, CDK-2, -4, -6* Migration inhibition *↓ Snail*	Apigenin synergizes with cisplatin significantly increasing its effects on prostate cancer stem cells (CSCs)	[57]
Apigenin	Fruits Vegetables Food herbs	Lung	Migration/invasion inhibition *↓ CD26/DPPIV* *↓ Akt, Snail/Slug EMT* Cell growth and metastasis inhibition *↓ CD26*	CD26^high^/Akt^high^ Tumors show the shortest recurrence times of non-small cell lung cancer. apigenin inhibits the migration/invasion of non-small cell lung cancer by targeting CD26	[58]
Curcumin	Turmeric	Prostate	Apoptosis *↓ Bcl-xl, NF-κB* Virus infection increase *↓ STAT1*	Curcumin synergizes with vesicular stomatitis virus modulating antiviral responses and potentiating components of the intrinsic apoptotic pathway.	[59]
Curcumin	Turmeric	Colorectal	Gene expression regulation in pathways related with DNA replication, cell cycle, protein export, glutathione metabolism and porphyrin metabolism *HSPA5, SEC61B, G6PD, HMOX1, PDE3B*	Cooperative mechanisms of action of curcumin and oligomeric proanthocyanidins show enhanced anti-tumoral properties, opening up new effective therapies.	[60]
Curcumin	Turmeric	Breast	Cell proliferation, migration, invasion suppression *↑ E-cadherin* *↓ Vimentin, Fibronectin,* *β-catenin* Decreased stem cell features *↓ Oct4, Nanog, Sox2*	Anti-metastasis activity of curcumin via the inhibition stem cell-like features and epithelial-mesenchymal transition.	[61]
Curcumin	Turmeric	Lung	Downregulated EGFR activity (growth inhibition) *↓ Sp1-HADC1 interaction* Signaling pathways inhibition *↓ RTKs, ERK/MEK, AKT/S6K* Autophagy induction	Combination of curcumin and gefitinib sensitizes EGFR-TKI resistance in wild-type EGFR and/or KRAS mutant cell lines promoting autophagy -mediated cell apoptosis.	[62]
Docosahexaenoic acid (DHA)	Fish or algae oils	Colorectal	Induced expression of genes related to apoptosis. Proteasome inhibition in favor of proapoptotic proteins resulting in an accumulation of tumor-suppressor proteins and induction of apoptosis.	DHA have chemopreventive effect significantly inhibiting the growth of cancer cells.	[63]
Docosahexaenoic acid (DHA)	Fish or algae oils	Colorectal	Inhibition of 5-FU-induced IL-1β secretion, caspase-1 activity, JNK activation	DHA enriched diet reduces circulating IL-1β concentration and recurrence in 5-FU-treated tumors	[64]
Epigallocatechin gallate (EGCG)	Green tea	Lung	Apoptosis *↑ GADD153, death receptor 5, and p21waf1* Protein acetylation inhibition *↓ HDAC4, -5, -6*	In combination with the synthetic retinoid Am80, EGCG or HDAC inhibitor celecoxib, enhances cell apoptosis and increases drug sensitivity in resistant cells.	[65]
Epigallocatechin gallate (EGCG)	Green tea	Lung	CSCs growth inhibition and apoptosis *↑ has-mir-485-5p* *↓ RXR*α	EGCG inhibits non–small-cell lung cancer cell growth and induces cell-apoptosis.	[66]
Epigallocatechin gallate (EGCG)	Green tea	Breast	Bioinformatic prediction: disruption of signaling proteins involved in cell death and survival, DNA replication, recombination and repair; and cell cycle *JUN, FADD, NFKB1, Bcl-2, GNAO1, MMP14*	EGCG is predicted to affect several molecular pathways that appear altered in breast cancer.	[67]
Epigallocatechin gallate (EGCG)	Green tea	Colorectal	Apoptosis and DNA damage *↓ GRP78, MDR1* *↑ NF-*κ*B, miR-155-5p*	EGCG acts as a chemo-sensitizer to 5-fluorouracil in colon cancer cell lines.	[68]
Naringenin	Citrus fruits	Prostate	Apoptosis *↑ PI3K/AKT* *↓ ERK1/2, p38, JNK* Loss of MMP ROS generation Loss of mitochondrial membrane potential	Naringenin suppresses cell proliferation and migration, and induces apoptosis and ROS production. In combination with paclitaxel, enhances cell proliferation inhibition effects.	[69]
Procyanidin B2 3,3″-di-O-gallate (B2G2)	Grape seed	Prostate	CSCs cell renewal *↓ Cleaved Notch1, HES-1,* *NF-κB, STAT3.*	B2G2 targets both differentiated cells and CSCs in the tumor mass and impairs prostate cancer growth and relapse	[70]
Quercetin	Fruits Vegetables Red wine	Prostate	Cell proliferation inhibition *↓ PI3K, AKT, ERK1/2, p38, ABCG2, NF-κB* Inhibition of migration in PC3 and CD44+/CD133+ *↓ PI3K/PTEN, MAPK,* *NF-κB*	Downmodulation of growth factor midkine (MK) expression curbs migration, tumorigenesis and progression of CD44+/CD133+ and prostate cancer cells. Quercetin enhances MK inhibition, promoting apoptosis and effectively eliminating cancer cells.	[71]
Quercetin	Fruits Vegetables Red wine	Breast	Cell proliferation inhibition *↓ mTOR, PI3K, Akt, CyclinD, Bcl-2* Cell viability inhibition *↓ ER*α	Quercetin inhibits PI3K/Akt/mTOR-signaling, decreasing proliferation in CD44+/CD24− CSCs, thereby decreasing breast CSC population.	[72]
Secoisolariciresinol diglucoside (SDG)	Flaxseed	Breast	Inhibition of tumor growth and macrophage infiltration Cell survival inhibition *↓ p65 and NF-κB*	SDG treatment, and in particular its metabolite enterolactone, correlates with restrained breast tumor growth in ERα-negative breast cancer. Therefore, SDS could be effective as an adjuvant treatment to reduce recurrence.	[73]
β-Sitosterol-d-glucoside (β-SDG)	Sweet potato	Breast	Activation of tumor supressors *↑ miR-10a* Cell signaling regulation *↓ PI3K/Akt, Bcl-2* Apoptosis *↑ caspase proteases*	Inhibitory effects of β-SDG breast-cancer cell growth. Promising therapeutic agent for treating breast cancer.	[74]

**Table 3:** Characteristics of included studies related to bioactive natural extracts.

Extract Source	Bioactive Fraction	Cancer Type	Molecular Mechanism	Anticancer Effect	Reference
Andrographis paniculata	Andrographolide	Prostate	Apoptosis Cell cycle and DNA repair modulation *ATM, BLM, BRCA2, BRIP1, CLSPN, NBN, PALB*	Andrographolide promotes DNA damage in tumor cells leading to cell death.	[75]
Aronia	3-O-p-Coumaroyltormentic Acid	Breast	Cell proliferation inhibition Reduction of cancer cell subpopulations *CD44^high^/CD24^low^, ALDH^+^* Self-renewal inhibition *↓ CD44, Sox2, Oct4* Cell survival inhibition *↓ c-Myc*	Promotes CSCs cell death inhibiting survival and self-renewal potential.	[76]
Castor oil	ω-hydroxyundec-9-enoic (ω-HUA)	Breast	Increased apoptosis and ROS generation *↑ Caspase-3, PARP, p38, JNK*	ω-HUA-induced cell death promotes tumor regression.	[77]
Ginger	Gingerols	Leukemia	Antiproliferative impact on methotrexate-resistant tumor cell lines not by modifying the expression levels of the *ABCA2* and *ABCA3* drug efflux genes.	Antitumor impact of ginger in combination with methotrexate on T-cell acute lymphoblastic leukemia (T-ALL).	[78]
Ginseng	Ginsenoside Rg3	Colorectal	Cell survival inhibition *↓ NF-*κ*B, Cyclin D1, Survivin, Cox-2, VEGF*	Rg3 enhances radiotherapy by impairing cell survival, finally inhibiting tumor growth.	[79]
Grape seed extract	Monomeric, dimeric and trimeric proantho-cyanidins (OPCs)	Colorectal	Cell cycle and DNA replication inhibition *↓ CCNE2, E2F1* *↑ SFN, CDKN1A, MAD1L1* Cell migration inhibition *↓ MMP2, EZH2, WNT5A* Upregulation tumor suppressor gene *PTEN*	OPCc block various oncogenic pathways and inhibit colorectal cancer growth through multiple cell pathways.	[80]
Isodon	Flexicaulin A	Colorectal	Cell proliferation inhibition *↑p21*	Flexicaulin A inhibits cancer cell proliferation, emerging as a promising support treatment in colorectal malignancies.	[81]
Orange peel	Nobiletin Sinensetin Sutellarein tetramethylether Tangeretin	Colorectal	Cell proliferation inhibition Cancer stemness and self-renewal inhibition *↓PROM1, LGR5* EMT transition modulation *↑CDH1* *↓ZEB1, SNAI1*	Orange peel extract reduces cell proliferation and modulating cancer stemness and self-renewal. Synergistical interaction with 5-fluorouracil.	[82]
Sorghum	Phenolic acids and flavonoids	Prostate	Apoptosis *↓ Bcl-2, Akt* *↑ Bax* Cell cycle arrest *↓ Cyclin D1, Cyclin E* *↑ p21Waf/Cip1*	Donganme sorghum ethyl- acetate extract (DSEE) suppresses cell proliferation by activating apoptosis.	[83]
Rosemary and shark liver oil rich in alkylglycerols	Phenolic diterpenes	Colorectal	Modulation of expression of genes involved in immune-modulation, inflammation, oxidative stress, lipid metabolism, and tumorigenesis.	Activation of innate immune, cytotoxic and anti-inflammatory responses towards effector cells. Gene expression modulation supports its potential usefulness in cancer patients.	[84]
Thunder god vine	Triptolide	Breast	Cell proliferation inhibition Caspase-3-mediated apoptosis Autophagy induction	Triptolide could be an efficient anticancer agent specific for triple negative breast cancers.	[85]
Watercress and broccoli extracts	Phenethyl isothiocyanate (PEITC) and sulforaphane (SFN)	Colorectal	Impaired cell proliferation Decreased cell self-renewal Decreased cell adhesion *↓ E-cadherin* Reversion of CSC ALDH1-mediated chemoresistance *↓ LGR5, PROM1, ALDH1* CSC proliferation *Wnt/β-catenin/TCF7L2*	Chemotherapeutic potential of ITC-enriched extracts in CRC therapy by targeting critical aspects of tumor progression and tumor relapse.	[86]

**Table 4:** Curcumin nano-formulations.

Bioactive Foodstuff	Cancer Type	Nano-Formulation	Molecular Mechanisms	Anticancer Effect	Reference
Curcumin	Breast	H-ferritin (HFn) nanoparticle	HFn biopolymer specifically binds to the TfR1 receptor, found to be overexpressed in triple negative breast cancer cells.	HFn nanoparticles raises solubility, stability and bioavailability of curcumin, potentiating its effects as a doxorubicin sensitizer.	[87]
Curcumin	Breast	Fe^3+^-curcumin and Cu^2+^-curcumin complexes encapsulated into poly(styrene)-co-maleic acid (SMA) micelles.	Metal complexes prevent curcumin degradation. Its sequential encapsulation into SMA micelles improves their solubility and stability and their accumulation in tumors.	Improved chemical stability and tumor growth reduction. Higher stability in biological fluids. Increased ability to enter and accumulate in tumor cells.	[88]
Curcumin	Prostate	Dextran nanobubbles	Effective internalization into tumor cells and sustained release of curcumin, enhancing curcumin potential to inhibit cell migration and promote apoptosis.	Lower doses of curcumin are needed to get the same anti-cancer effects. Helping to prevent metastasis and relapse.	[89]
Curcumin in combination paclitaxel	Breast	Hyaluronic acid (HA) lipoid hybrid nanoparticles	HA interacts with the CD44 receptor, overexpressed in breast CSCs.	Enhanced anti-tumor impact by inhibiting cell growth and migration.	[90]
Curcumin in combination paclitaxel	Breast	Poly (ethylene glycol)-benzoic imine-poly(g-benzyl-L-aspartate)-b-poly(1-vinylimidazole) block copolymer	This pH polymer can switch its surface charge in order to facilitate their intake by tumor cells, solving issues regarding drug delivery into inner regions of solid tumors.	The formulation increases the extent of action of the curcumin-paclitaxel combination.	[91]
